# Dairy calves’ personality traits predict social proximity and response to an emotional challenge

**DOI:** 10.1038/s41598-018-34281-2

**Published:** 2018-11-05

**Authors:** Benjamin Lecorps, Sarah Kappel, Daniel M. Weary, Marina A. G. von Keyserlingk

**Affiliations:** Animal Welfare Program, Faculty of Land and Food Systems, University of British Columbia, 2357 Main Mall, Vancouver, BC V6T 1Z6 Canada

## Abstract

The assessment of individual traits requires that tests are reliable (i.e. consistency over time) and externally valid, meaning that they predict future responses in similar contexts (i.e. convergent validity) but do not predict responses to unrelated situations (i.e. discriminant validity). The aim of this study was to determine if dairy calf personality traits (Fearfulness, Sociability and Pessimism), derived from behaviours expressed in standardized tests, predict individuals’ responses in related situations. The first experiment tested if the trait ‘Sociability’ was related to the expression of social behaviour in the home-pen, with calves assigned individual proximity scores (based on proximity to other calves) while they were in their home-pen at approximately 113 and 118 d of age. The second experiment aimed at exploring whether traits ‘Fearfulness’ and ‘Pessimism’ were related to the calves’ emotional response to transportation. All calves were subjected to two 10-min transportation challenges done on two consecutive days. Emotional response was assessed using the maximum eye temperature (measured using infrared thermography) and the number of vocalizations emitted. Social proximity scores (Experiment 1), vocalizations emitted and maximum eye temperature after loading (Experiment 2) were consistent over time. In addition, the results showed good convergent validity with calves scoring higher in Sociability also having higher proximity scores in the home-pen, and animals scoring higher in Fearfulness and Pessimism showing a more intense emotional response to transportation. The results also showed good discriminant validity, as neither Fearfulness nor Pessimism were associated with the expression of social behaviours (Experiment 1) and Sociability was not associated with the animal’s emotional response to transportation (Experiment 2). We conclude that the methodology used to measure personality traits shows good reliability and external validity.

## Introduction

Individual traits, often referred to as personality traits, are defined as behaviours or suites of behaviour that consistently vary among individuals. One recent framework describes five main traits: shyness-boldness, exploration-avoidance, activity, sociability and aggressiveness^1^. Among these, the most commonly studied are based on how animals react to emotionally challenging situations or to potential threats (e.g. shyness/boldness, or in case of farm animals, fearfulness) and the way animals behave with their social partners (e.g. sociability, aggressiveness). Fearfulness can be seen as a “general susceptibility to react to a variety of potentially threatening situations”^2^, with bolder individuals more prone to take risks while shyer ones are considered more careful^3^, and Sociability refers to the propensity of an individual to keep close contact with conspecifics^1^. Recent work in farm animals showed stable individual differences for both traits^4–6^.

Personality traits are typically identified using standardized behavioural tests^7^, although difficulties remain in understanding behaviours expressed during these tests^8^. In farm animals, assessment of personality traits has mainly relied on open field, novel object, and human reactivity tests^7,9^. However, few studies have addressed whether these tests predict responses in more naturalistic situations (i.e. their validity^7,8^). As argued by Perals *et al*. (2017), personality traits labelled through the use of standardized tests need to be validated using behaviours expressed in related contexts^10^. Ideally, a trait predicts individual responses in related situations (i.e. shows convergent validity) but not in unrelated ones (i.e. shows discriminant validity). For instance, Fearfulness should be able to predict behaviours that are expressed in fearful situations but not in others.

The aim of the current study was to assess the validity of the dairy calf personality traits described in a previous study (i.e. Pessimism, Fearfulness and Sociability)^6^. These traits were identified using responses to standardized tests (Pessimism as assessed using repeated judgment bias tests; Fearfulness and Sociability as assessed using open field, novel object, human reactivity and social motivation tests). Similar behaviours measured in the different tests were aggregated as this generates more reliable information than single-context measures^11–13^. Calves were characterized at approximately 25d and 50d old and all three traits were individually consistent across two test sessions. Furthermore, Pessimism and Fearfulness were positively related, suggestive of a behavioural syndrome^14^. To assess both convergent and discriminant validity, we measured individual responses in two different situations. In Experiment 1, individual proximity scores were calculated in the home-pen. We expected that more sociable animals (but not more fearful or pessimistic animals) would have higher social contact with the others. In Experiment 2, we measured the behavioural (i.e. vocalizations) and physiological (maximum eye temperature) response to transportation, a known stressor for farm animals^15^. We expected that Fearfulness and Pessimism (but not Sociability) would relate to the intensity of the stress response.

## Methods

These experiments were conducted at the University of British Columbia’s (UBC) Dairy Education and Research Centre, located in Agassiz, British Colombia, Canada (49°N, 121°W). The animals were cared for according to the guidelines outlined by the Canadian Council of Animal Care (2009). All procedures carried out in this study were approved by the UBC Animal Ethics Committee (A15–0117). All methods were performed in accordance with the relevant guidelines and regulations.

### Animals and housing

We used 17 of the 21 animals tested by Lecorps *et al*. (2018), and added two additional animals housed in the same groups providing a sample of 19 calves. The four animals from Lecorps *et al*. (2018) that were not included were housed in another group that could not be subjected to the transportation treatment of the current study. Holstein calves were reared according to standard practice at the UBC Dairy Education and Research Centre. Calves were group-housed from 5 d of age. Before weaning, animals had access to 12 L of pasteurized milk as well as *ad libitum* access to grain, hay and water (see^6^ for additional details). Calves were weaned gradually by decreasing the amount of milk calves had access to from 12 to 0 L a day over a 2-week period ending at approximately 100 d of age. At the time of testing for the current study (when calves were approximately 4 months old), animals were group housed (n = 9 ± 1 heifers per group) on an open, sawdust-bedded pack measuring 7 × 5 m. Calves had *ad libitum* access of grain (Hi-Pro Medicated Calf Starter, Chilliwack, BC, Canada with an overall DM of 89.5%; chemical composition shown as % of DM, 90% DM; CP 21%, NDF 19%, ADF 11%), hay and water.

### Individual traits

A full description of the assessment of Pessimism, Fearfulness and Sociability can be found in Lecorps *et al*. (2018). Briefly, animals were tested for judgment bias using a spatial learning task similar to that used on sheep^16^. Testing was conducted at 25 and 50 d of age. In both sessions, calves were presented with an empty bottle placed at three ambiguous locations located between the previously rewarded and punished training locations. We used the average latency to reach ambiguous locations as a measure of Pessimism.

Fearfulness and Sociability were assessed using four standardized tests (Open field, Novel object, Human reactivity and Social motivation test), commonly used in farm animals^4,7^. One test was performed per day.

### Home-pen social behaviour

Social behaviours were assessed when the animals were 113 ± 8.3 and 118 ± 8.3 d of age, approximately 30 d after weaning off milk. Behaviours were recorded using two digital cameras (WV-CW504SP, Panasonic, Osaka, Japan) placed 5 m above the calves for two periods of 48 h (T1 and T2).

Calf behaviour (lying or standing) and position relative to any neighbouring calves were recorded using 5-min scan sampling for two sessions of 48 h separated by 3 d. Scans in which animals were feeding were excluded to avoid confounding proximity with competition for food. To be considered neighbours, animals were required to be less than one head length apart^17^. Individual proximity scores were calculated as follows:

$${\rm{Individual}}\,{\rm{proximity}}\,{\rm{score}}=\frac{Number\,of\,neighbours\,while\,resting}{Number\,of\,scans\,resting}+\frac{Number\,of\,neighbours\,while\,standing}{Number\,of\,scans\,standing}$$

### Transportation

Each calf was individually transported on two 10-min journeys undertaken on consecutive days when they were approximately 120 ± 8.3 d of age. Calves were tested in random order and one at the time. The calf was gently moved out of the group by a familiar handler and loaded onto an open trailer (140 × 120 × 110 cm). After loading, the trailer remained stationary for 1 min, was then towed by a vehicle for 5 min, was again stationary for 1 min and then again towed for 5 min. The trailer then remained stationary for an additional 1 min before unloading. Total distance was 2 km of paved road including 12 turns at an average speed of 12 km/h and never exceeding 20 km/h. Although, the configuration of the trailer prevented animals from jumping out, the vehicle was immobilized in the rare cases where animals fell inside the trailer (2 times over 38 transportations), giving them sufficient time to stand up before transportation continued.

### Physiological and behavioural measurements

Maximum eye temperature was measured with an infrared camera (T650sc, FLIR Systems USA, Boston, MA). Distance between the camera and the eye was kept constant at approximately 1 m. Measurements were taken at three time points: 1) 30 min before any kind of perturbation (Baseline), 2) just after loading in the trailer (Post-loading), and 3) at the end of transportation (Post-transportation). Video files were analysed with the FlirResearchIR software (Flir Systems USA, Boston, MA). For each picture, the ambient temperature at the time of transportation was set in the software during the analysis. A minimum of 1 and a maximum of 5 images were taken for each time point. Images were analysed only if they provided a clear and focused view of the eyes. Baseline values were used to calculate changes in maximum eye temperature after loading and after transportation. In addition, as maximum eye temperature measured at the beginning of unconditioned fear tests were recently shown to predict behavioural phenotype^18^, we also used maximum eye temperature after loading for further analyses. In addition, we counted the number of vocalizations from the moment the calf entered the trailer until it was unloaded.

### Statistical analysis

All analyses were done with R (version 3.4.2). Linear regressions were calculated using the *lm* function and the associated p-value was calculated via permutation (Package pgirmess^19^). From the 19 animals used in the study 17 had been phenotyped for personality traits earlier in life. Consequently, when referring to personality traits, the results of this study are based on these 17 animals only.

### Personality traits

Principle component analysis was used to define the personality traits, as described in Lecorps *et al*. (2018).

### Social proximity score

We calculated an individual proximity score for each of the two 48 h sessions. Consistency over time was assessed using linear regression. We then averaged the proximity scores over the two time-periods to get one measure per calf and assessed the relationship between this measure and each of the personality traits.

### Emotional response to transportation

When multiple pictures were available and fit the criterion (range: 1–5), we averaged eye temperature obtained to provide one measure of maximum eye temperature for the baseline, the post-loading and the post-transportation time-points. Changes in maximum eye temperature in relation to baseline values and maximum eye temperatures were used for the statistical analysis. The total number of vocalizations during the 10 min of transport were live-recorded. As two cohorts of calves were transported on different days with different weather conditions, we included group as an interactive term in all models to control for ambient temperature differences. We assessed consistency over time for all measures (i.e. over the two transportation challenges). For the rest of the analysis we used the averages for each measure taken during both transportations. Consistency over time, the relationship between behavioural and physiological measures, and the relationships with the different individual traits were all assessed using a linear model with *P* values extracted using a non-parametric permutation approach as described above.

## Results

### Experiment 1: Home-pen social behaviour

**Figure 1:**
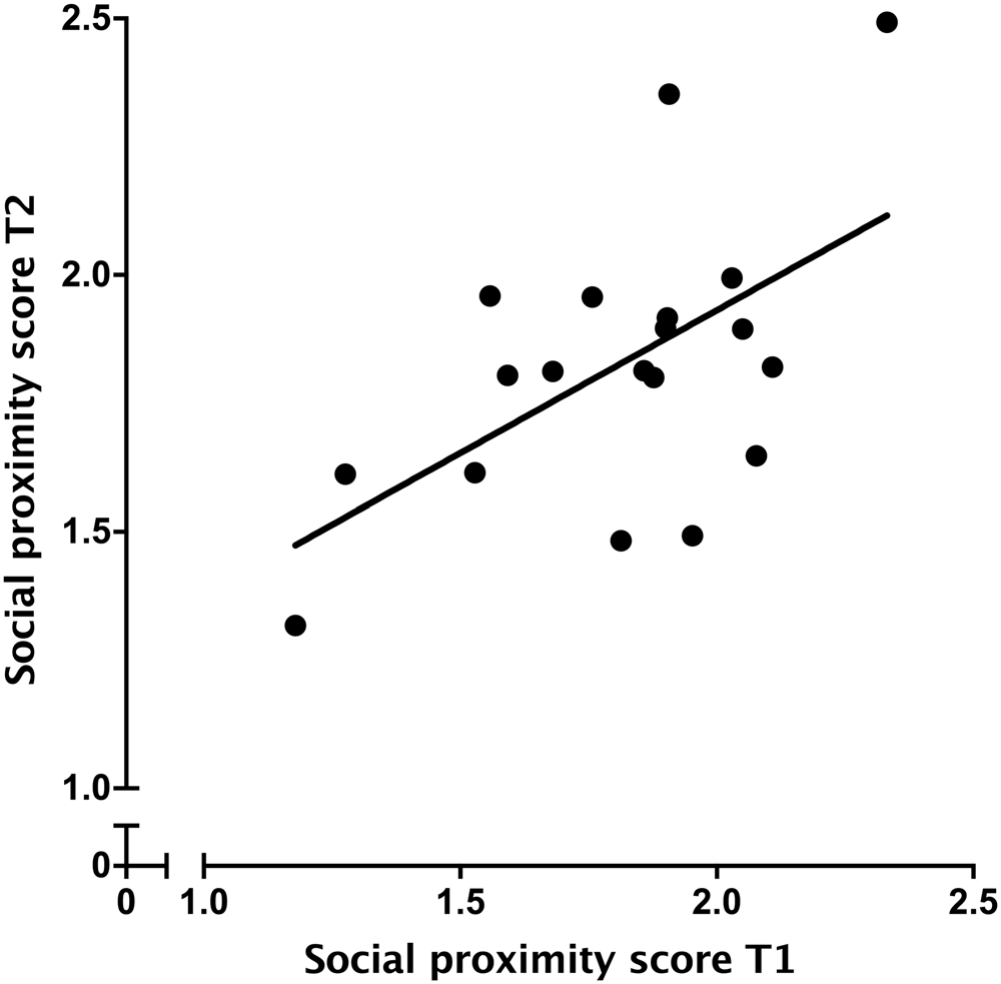
Consistency in individual social proximity scores of Holstein heifers (n = 19) tested twice within 1 week at approximately 115 days of age. Proximity scores were calculated using the total number of neighbours each calf had during each 48-h session, divided by the number of scans in which the calf was either lying or standing.

Calves showed consistency in their proximity score across the two-observational periods (*R*^2^ = 0.33, *P* = 0.009; Fig. 1); animals that had more neighbours in the first period also had more neighbours in the second.

**Figure 2:**
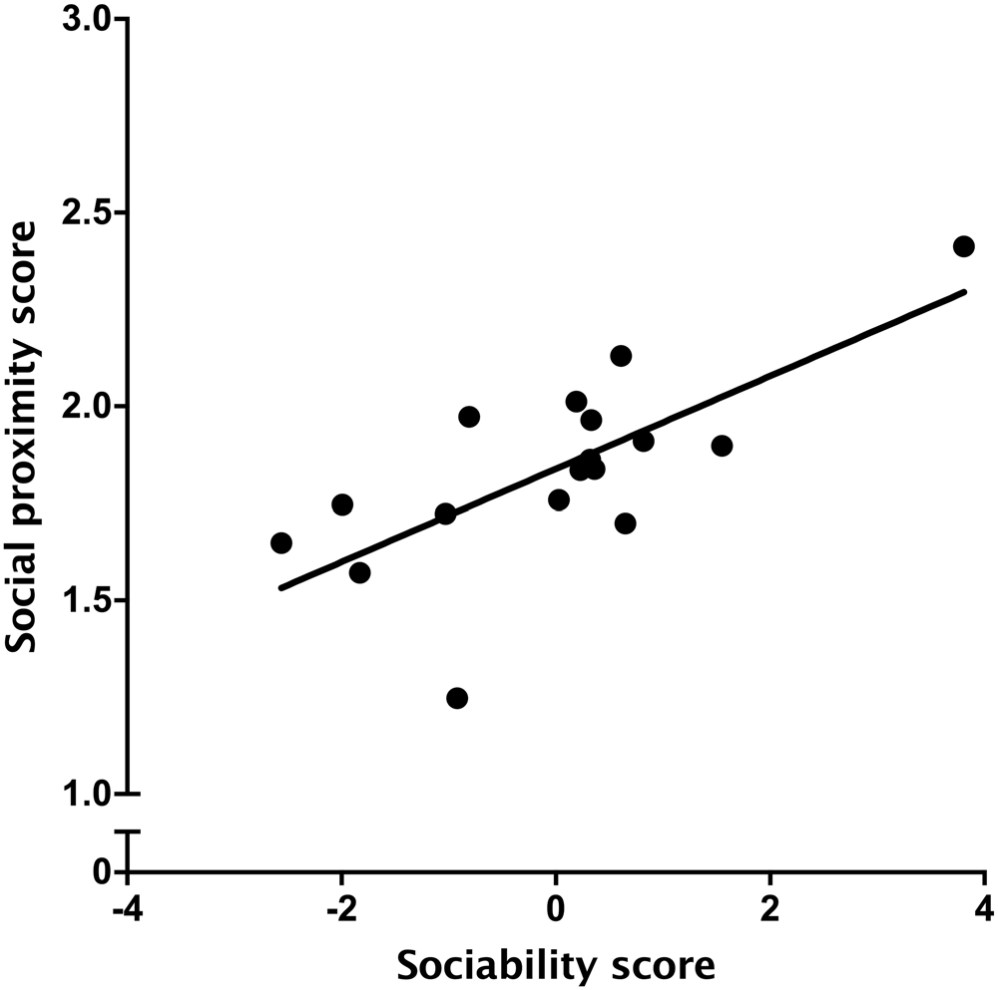
Relationship between calf Sociability (from the personality assessment done when the animals were approximately 1 and 2 months old) and social proximity score from home-pen observations (n = 17). The proximity score was calculated by averaging measures over the 4 d of observations (at approximately 115 d old). The Sociability trait was derived from principal component analysis using responses to four standardized tests; higher values correspond to higher levels of Sociability.

The average social proximity score was positively related to the Sociability trait derived from the personality assessment (*R*^2^ = 0.51, *P* = 0.002, Fig. 2), with animals scoring higher on the Sociability trait having higher proximity scores in their home pen.

### Experiment 2: Emotional response to transportation

**Figure 3:**
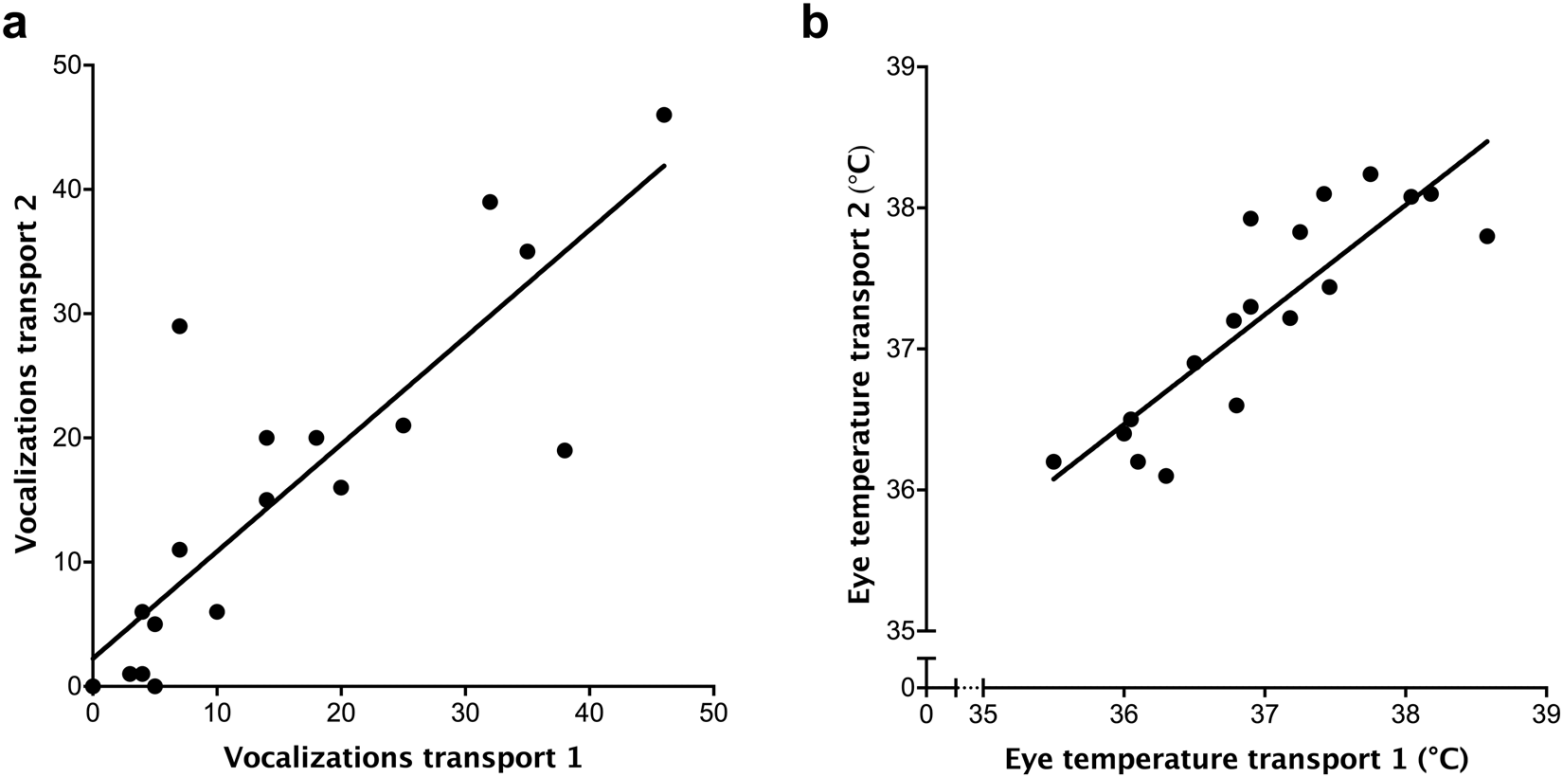
Consistency across two consecutive days for (**a**) the number of vocalizations expressed by dairy heifers during 10 min transportation events, and (**b**) the maximum eye temperature of each animal measured after loading. Each point represents a calf (n = 19).

Calves were consistent over the two transportations in their vocal responses (*R*^2^ = 0.76, *P* < 0.0001; Fig. 3a) and maximum eye temperature measured after loading (*R*^2^ = 0.76, *P* < 0.0001; Fig. 3b) and after transportation (*R*^2^ = 0.64, *β* = 0.032, *P* = 0.0013). Changes in maximum eye temperature from baseline to loading and transportation were not found consistent (*P* > 0.05).

**Figure 4:**
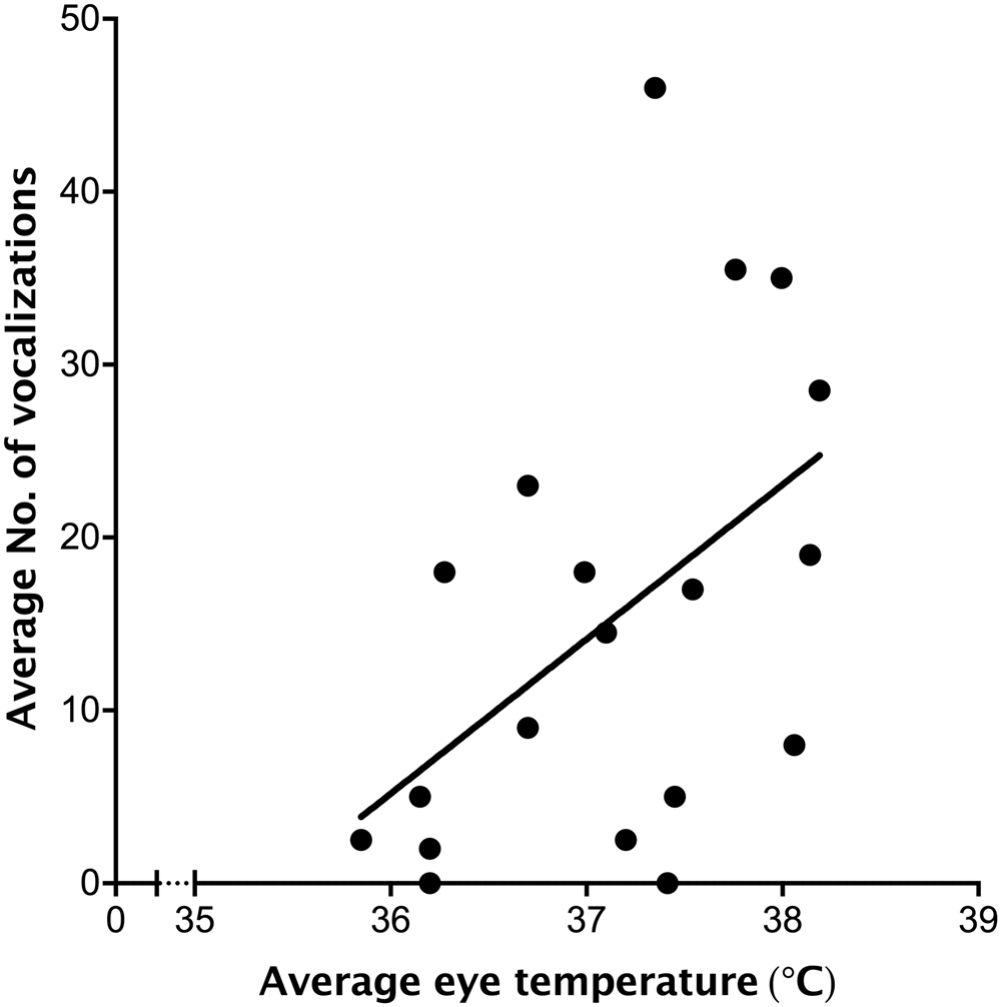
Relationship between the maximum eye temperature measured after loading and the number of vocalizations by dairy heifers during transport when they were 4 months old. Measures were averaged over the two transport sessions completed on consecutive days. Each point represents a calf (n = 19).

The average number of vocalizations during transport was positively related to the average maximum eye temperature after loading (*R*^2^ = 0.38, *P* = 0.028; Fig. 4) but not to the maximum eye temperature after transport (*P* > 0.05). Similarly, vocalizations were not related to the change in maximum eye temperature for either period (*P* > 0.1).

**Figure 5:**
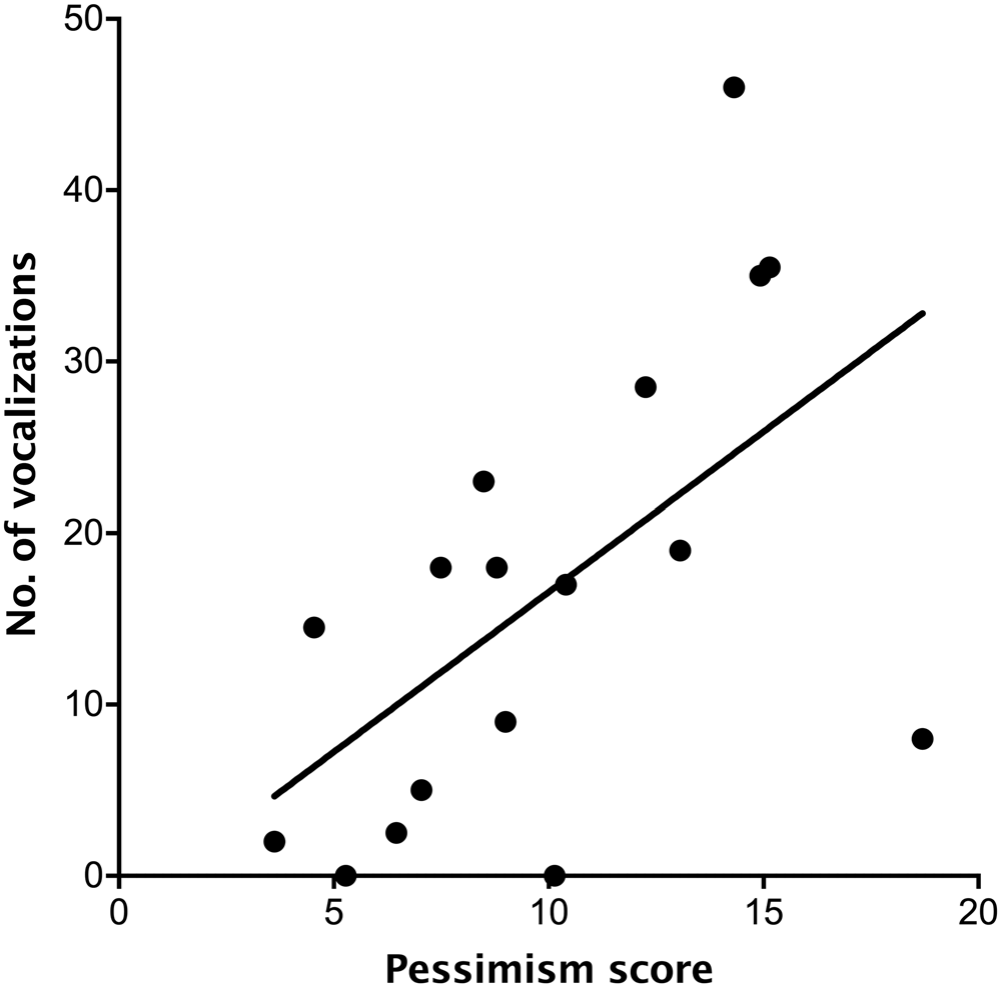
Relationship between the trait Pessimism (assessed when dairy heifer calves were approximately 1 and 2 months old) and the average number of vocalizations expressed (over two transportation challenges completed on consecutive days when the animals were approximately 4 months old). Each point represents a calf (n = 17).

**Figure 6:**
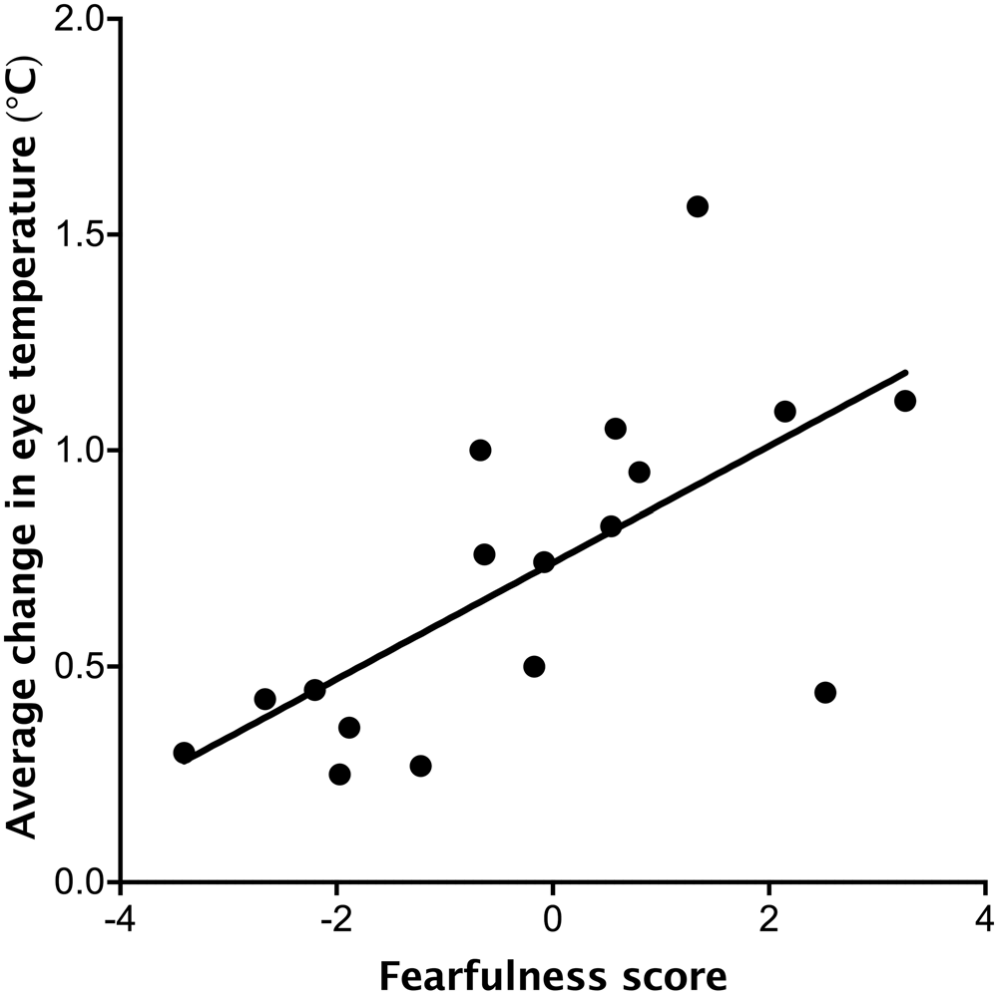
Relationship between the trait Fearfulness (assessed when the calves were approximately 1 and 2 months old) and the change in maximum eye temperature after loading into the trailer (when calves were 4 months old). Each point represents a calf (n = 17).

The average number of vocalizations during transport was not related to Fearfulness or Sociability (*Ps* > 0.05) but was positively related to Pessimism (*R*^2^ = 0.46, *P* = 0.014; Fig. 5). The average post-loading change in maximum eye temperature was positively related to Fearfulness (*R*^2^ = 0.56, *P* = 0.0022; Fig. 6) but not to Pessimism or Sociability (*P* > 0.05).

**Figure 7:**
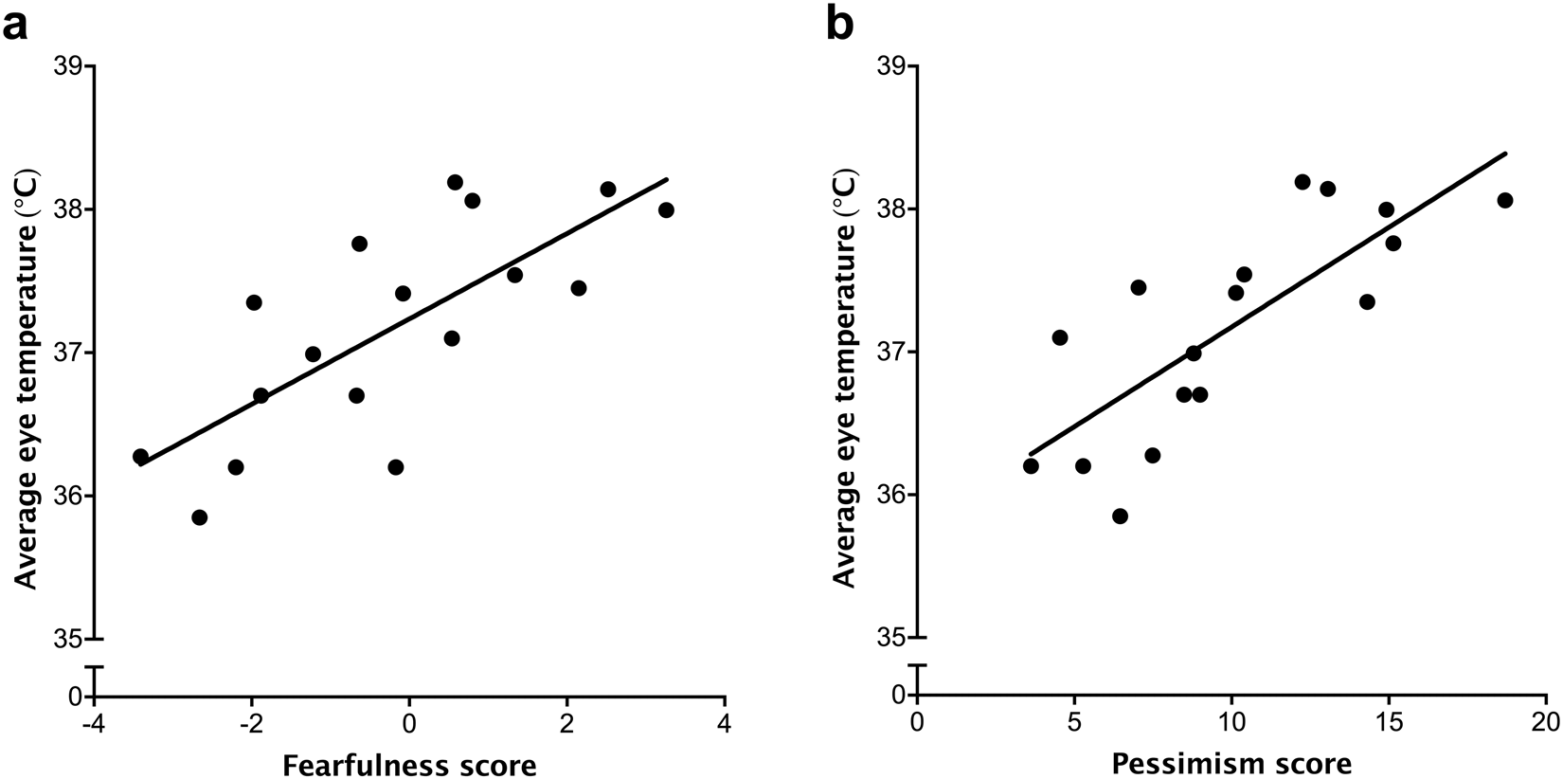
Relationship between traits (**a**) Fearfulness and (**b**) Pessimism (assessed when calves were approximately 1 and 2 months old) and the average maximum eye temperature measured after loading into the trailer (assessed when calves were about 4 months old). Each point represents a calf (n = 17).

The average post-transportation changes in maximum eye temperature were not related to any of the three personality traits (*P* > 0.05), but average post-loading maximum eye temperature was positively related to Fearfulness (*R*^2^ = 0.72, *P* < 0.001; Fig. 7a) and Pessimism (*R*^2^ = 0.75, *P* < 0.001; Fig. 7b), but not Sociability (*P* > 0.05).

## Discussion

Poor validity is a major risk in personality studies and is associated with mislabeling and misinterpretation of behaviours expressed in personality tests^8^. Personality traits should both be consistent over time (i.e. reliability), and also show good external validity. The aim of this study was to investigate whether personality traits assessed during the two first month of life were valid predictors of social behaviour in the home-pen and emotional responses to transportation, tested when the animals were about 4 months old. All three personality traits showed good convergent and discriminant validity. Sociability was positively related to social proximity scores in the home-pen but was not related to the way animals responded to transportation. Fearfulness and Pessimism were predictors of the response to transportation (with more fearful and pessimistic animals showing elevated signs of distress), but not of social behaviours expressed in the home-pen.

The trait Sociability has been described in many species^1^ and can be assessed in different ways. More sociable individuals are expected to seek the presence of conspecifics, while less sociable individuals should avoid them. Thus, Sociability tests have focused on how much animals aggregate with others^20^, react to separation^21–24^ or are motivated to maintain close proximity^4^. Some have also assessed how animals behave during a forced interaction, with more sociable animals expected to display more pro-social and less aggressive behaviours. All of these different tests give opportunities to measure convergent validity of Sociability. In our previous study, Sociability was defined by how animals reacted to social isolation (i.e. number of vocalizations emitted) and by their motivation to return to the herd in the runway test; responses that were found highly stable over time^6^. In the current study, proximity scores were also found to be consistent over time, with some animals spending more time, and others less time, at close proximity with other calves. We hypothesized that more sociable animals, as defined in the personality tests, would also seek close contact with their conspecifics in the home-pen. Our results showed that more sociable animals had higher proximity scores indicating good convergent validity of the Sociability trait. Similar results have been found in felids^25^, sheep (*Ovis aries*)^24^ and dogs (*Canis familiaris*)^26^ as well as in adult dairy cattle (where motivation to join the herd after isolation was positively related to the number of social neighbors and synchronicity with the group^4^). Our results indicate both convergent and discriminant validity, as Sociability was related to social proximity but not to the way animals reacted to transportation.

Fearfulness and its counterpart boldness are usually measured during tests where animals are exposed to different forms of novelty (e.g. novel environment or object). Some previous reports have found Fearfulness – measured using standardized behavioural tests – to be inconsistent over time and to have poor convergent or discriminant validity (Japanese Quail (*Coturnix japonica*)^27^, Damselfish (*Pomacentrus wardi* and *P. amboinensis*)^28^, African striped mouse (*Rhabdomys pumilio*)^29^), but others have reported high degrees of consistency over time and also good external validity (Dogs^30^, Vervet Monkeys (*Chlorocebus pygerythrus*)^31^, African striped mouse^32^). Our results are consistent with the latter studies as calves responded consistently when subjected to two transportation events done on consecutive days and Fearfulness (assessed 2 months before the transportation event) reliably predicted the way animals responded to transportations. Indeed, Fearfulness predicted the change in maximum eye temperature taken before and after loading into the trailer with more fearful animals having higher increases indicating a more intense stress response. Work on horses (*Equus ferus*) showed that changes in maximum eye temperature were related to behaviours expressed in a novel object test; more fearful animals showing greater changes in maximum eye temperature^33^. In addition, the trait Fearfulness predicted maximum eye temperature measured immediately after loading into the trailer. In mice (*Mus musculus*), maximum eye temperature taken at the beginning of an open-field and elevated plus maze tests predicted the amount of anxiety-related behaviours expressed during the tests^18^. Collectively these results suggest that fearful animals react more strongly to pre-experimental handling causing higher eye temperatures at the beginning of the tests compared to their non-fearful counterparts. Contrary to our prediction, Fearfulness failed to predict the number of vocalizations emitted during transportation, a validated measure of distress^34^ and high arousal^35^.

Although judgment biases are now extensively used to assess emotional states of animals, some recent studies have shown animals to be consistent in the way they judge ambiguous situations, defining Pessimism as a trait^6,36^. In this study, the trait Pessimism was positively related to maximum eye temperature after loading in the trailer and to the number of vocalizations expressed during transport. These results indicate that Pessimism can predict both behavioural and physiological responses to an emotional challenge such as transportation. To our knowledge, our study is the first to investigate whether individual differences in judgment bias are related to an emotional response elicited by a challenging situation later in life (in our case more than 70 days after the last judgment bias test). These results suggest that more pessimistic animals react more intensely to the emotional challenge, maybe because they had worse expectations than other calves. These results are consistent with the human literature showing that pessimistic people react more strongly to emotional challenges^37,38^.

Collectively, our results indicate that the personality traits showed a good degree of validity and measured the targeted traits. Given that personality assessment is usually restricted to behavioural testing in animals, it is critical that traits are subjected to further validation, including testing of animals’ responses to more natural situations with clear predictions about how they should affect animal’s responses. Future studies should confirm our findings and if possible make use of other methodologies. For instance, studies exploring how affect manipulations (using pharmacological agents) modulate behaviours expressed in fear tests are still lacking in farm animals. In addition, the validity of Pessimism as a trait is still in its infancy in animal species and future studies should aim at understanding how it modulates an animals’ life. We see much room for work that is based on predictions arising from the wealth of information available from the human literature. As suggested by Nettle & Penke (2010), specific personality types are likely to react to sets of situations in specific ways^39^. For instance, neurotic persons show a greater physiological stress response when subjected to challenges^40^. We expect that pessimistic and fearful animals might react strongly to all emotional challenges and be more negatively affected by routine farm procedures. Recent results on laboratory rats (*Rattus norvegicus*) showed that more pessimistic animals are more sensitive to stress-induced anhedonia^41^, experience higher motivational loss after chronic stress^36^ and have a more compromised immune system^42^. Taken together this evidence suggests that these animals are at higher risk of developing depressive states or poor psychological welfare. Future studies should evaluate whether Fearfulness and Pessimism can predict animal’s response to other type of emotional challenges and how these traits affect the welfare of the animals in the longer term.

## Conclusion

Three personality traits (Pessimism, Fearfulness and Sociability) determined using standardized behavioural tests during the first two months of life in dairy calves showed good convergent and discriminant validity. More sociable animals had higher home-pen individual proximity scores. In contrast, more fearful and pessimistic animals showed a stronger emotional response to transportation.

## Electronic supplementary material

Dataset 1

## Data Availability

The data set from this study can be found in the supplementary material.
